# Identification and cost of adverse events in metastatic breast cancer in taxane and capecitabine based regimens

**DOI:** 10.1186/2193-1801-3-259

**Published:** 2014-05-21

**Authors:** Ryan N Hansen, Scott D Ramsey, Deepa Lalla, Anthony Masaquel, Tripthi Kamath, Melissa Brammer, Sara A Hurvitz, Sean D Sullivan

**Affiliations:** University of Washington, Seattle, WA USA; Fred Hutchinson Cancer Research Center, Seattle, WA USA; Genentech Inc, South San Francisco, CA USA; UCLA/Jonsson Comprehensive Cancer Center, Los Angeles, CA USA; Pharmaceutical Outcomes Research and Policy Program, School of Pharmacy, University of Washington, 1959 NE Pacific Ave, H-375Q, Box 357630, Seattle, WA 98195-7630 USA

**Keywords:** Breast neoplasms, Adverse effects, Antineoplastic agents, Costs and cost analysis

## Abstract

**Purpose:**

We sought to compare the economic impact of treatment-related adverse events (AEs) in patients with metastatic breast cancer (mBC) using taxane- or capecitabine-based treatment regimens as either first- or second-line (FL or SL) therapy in the US.

**Methods:**

We used healthcare claims data from the Truven Health Analytics MarketScan® Commercial Databases to conduct a retrospective cohort study comparing the economic impact of AEs amongst taxane- and capecitabine-treated mBC patients in the US. We selected women diagnosed with mBC between 2008–2010 who received a taxane or capecitabine as first- or second-line (FL or SL) chemotherapy. Costs related to hospitalization, outpatient services, emergency department visits, chemotherapy and other medications were tabulated and combined to determine total healthcare costs. The incremental monthly costs associated with the presence of AEs compared to no AEs were estimated using generalized linear models, controlling for age and Charlson Comorbidity Index.

**Results:**

We identified 15,443 mBC patients meeting inclusion criteria. Adjusted total monthly costs were significantly higher in those who experienced AEs than in those without AEs in both lines of treatment (FL incremental cost: taxanes $1,142, capecitabine $1,817; SL incremental cost: taxanes $1,448, capecitabine $4,437). Total costs increased with the number of AEs and were primarily driven by increased hospitalization amongst those with AEs.

**Conclusions:**

Adverse events in taxane- or capecitabine-treated mBC patients are associated with significant increases in costs. Selecting treatment options associated with fewer AEs may reduce costs and improve outcomes in these patients.

## Introduction

It is estimated that over 155,000 women are currently living with metastatic breast cancer (mBC) in the United States (US) (Metastatic Breast Cancer Network 2010). Treatment for mBC is generally considered palliative in intent, and the primary goals are to prolong survival while improving symptoms and preserving quality of life (Beslija et al. 2009). Selecting amongst available treatment options is complex and depends upon numerous factors, such as patient preferences and characteristics, comorbidities, symptoms, location/extent of metastases, history of prior treatments, and tumor markers (Beslija et al. 2009; National Comprehensive Cancer Network 2013). In addition, potential treatment-related side effects should be considered (Beslija et al. 2009). Chemotherapy-related adverse events (AEs) are common, and toxicity profiles vary across agents. Adverse events impact patient quality of life and can result in treatment interruptions or dose reductions, which in turn may decrease treatment efficacy (Kayl and Meyers 2006; Hwang et al. 2013). Furthermore, chemotherapy-related AEs can result in substantial increases in costs (Guerin et al. 2011; Hurvitz et al. 2012; Chu et al. 2009; Stokes et al. 2009; Craver et al. 2011).

The National Comprehensive Cancer Network Clinical Practice Guidelines (NCCN Guidelines) recommend various chemotherapeutic agents, used either as monotherapy or in combination, to prolong survival in patients with mBC (National Comprehensive Cancer Network 2013). Taxanes, such as paclitaxel and docetaxel, are commonly used. The NCCN guidelines list paclitaxel as a preferred single agent and docetaxel as an alternative single agent in the treatment of mBC (National Comprehensive Cancer Network 2013). Both of these agents are administered intravenously and may be used alone or as a part of a combination regimen. Capecitabine, an orally administered antimetabolite, is also listed by the NCCN guidelines as a preferred single agent in the treatment of mBC (National Comprehensive Cancer Network 2013). However, the cost of AEs related to capecitabine and taxanes in mBC patients is not well understood. An earlier analysis investigating this issue used the PharMetrics® Integrated Database to analyze healthcare costs amongst patients with mBC receiving a taxane or capecitabine and found that chemotherapy-related AEs were associated with a substantial economic burden, driven by increased inpatient, outpatient, and pharmacy costs (Hurvitz et al. 2012).

The current study investigates a similar question, using a different healthcare claims database. The results of this study will serve as a validation of previous work, using a different methodology. Our objective was to compare the economic impact of treatment-related AEs in patients with mBC using taxane- or capecitabine-based treatment regimens as either first- or second-line (FL or SL) therapy in the US.

## Methods

### Design and data source

**Figure 1:**
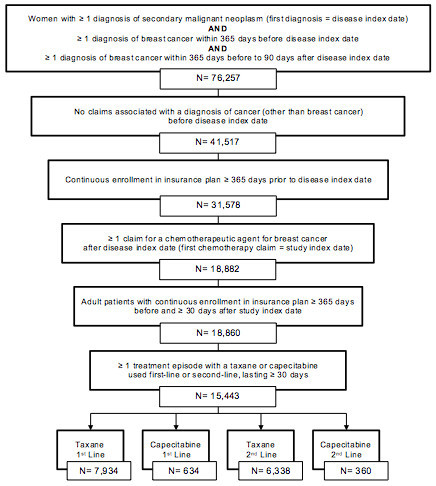
**Flowchart of sample selection.**

We conducted a retrospective cohort study among patients with mBC treated with a taxane or capecitabine in the first or second line setting. Healthcare claims data from the Truven Health Analytics MarketScan® Commercial Databases were used to identify line of treatment and to estimate AE frequency and costs. The MarketScan database includes comprehensive claim-level data on inpatient, outpatient, and prescription drug claims for over 90 million employed individuals and their dependents in the US. Claims data are also matched to administrative records, which provide demographic information and monthly insurance coverage status, allowing for longitudinal tracking of individuals.We selected women with claims indicating a diagnosis of secondary malignant neoplasm (International Classification of Diseases version 9 [ICD-9] 196.X-198.X) occurring between 2008–2010 (Figure 1). The first metastatic cancer diagnosis was set as the “disease index date.” In order to restrict the sample to those with metastatic breast cancer, we required that patients have: (1) at least one claim with a breast cancer diagnosis (ICD-9 174.X, 233.0, 85.20–85.23, 85.41–85.48) within the 365 days prior to disease index, and (2) at least one additional claim with a breast cancer diagnosis, as defined above, within 365 days prior to or 90 days after disease index. In addition, patients were excluded if they had any claims for a cancer other than breast cancer (defined as ICD-9-CM 140.x-165.x, 170.x-173.x, 7 175.x-176.x, 179.x-195.x, and 199.x-209.x) prior to disease index. Additional eligibility criteria included: (1) continuous insurance coverage for one year prior to and one year after disease index, (2) at least one claim for a chemotherapy drug for breast cancer after disease index, (3) continuous insurance coverage for at least one year prior to and 30 days after the first chemotherapy treatment, and (4) at least one treatment episode with a taxane (paclitaxel or docetaxel) or capecitabine as FL or SL therapy.

The date of treatment initiation with a chemotherapeutic agent, following disease index, was designated the “study index date”. Patients were followed longitudinally, using pharmacy dispensing and medical claims data to determine each patient’s course of treatment. The follow-up period began at study index and ran until the end of continuous health plan enrollment or the end of data availability, whichever came first. This period included one or more treatment episode(s) with a taxane or capecitabine. A treatment episode was defined as the period from treatment initiation until a gap in treatment of over 45 consecutive days or a change in regimen (switching medications, adding a new medication, or discontinuing a medication). Only treatment episodes lasting at least 30 days were included in the analysis. For each treatment episode, eligible patients were categorized into one of four treatment cohorts: (1) first-line taxane, (2) first-line capecitabine, (3) second-line taxane, or (4) second-line capecitabine. Episodes were excluded in which patients received both a taxane and capecitabine, but patients could use each agent during separate treatment episodes. Patients were assigned to treatment cohorts using a published algorithm, which incorporates both pharmacy and medical claims data (using Health Care Procedure J-codes and National Drug Codes) (Hurvitz et al. 2012).

Chemotherapy-related AEs were identified in the inpatient and outpatient service-level data, based on ICD-9 codes (Table 1). The list of investigated AEs was developed based on a combination of published data, package inserts and clinical opinion (Hurvitz et al. 2012). Adverse events were attributed to the treatment received during the episode in which the diagnosis of the AE occurred. For each episode, patients were categorized based on the total number of AEs they experienced during that episode (0, 1–2, 3–4, or > 4 AEs).

**Table 1:** **Diagnosis codes for adverse event identification**

Adverse event type	ICD-9 code(s)
Anemia	281, 283, 284, 285
Arthralgia	719.4x
Elevated Bilirubin	277.4
Constipation	546.0x
Diarrhea	007, 009.x, 787.91, 564.5
Dyspnea	786
Edema	782.3
Fatigue	780.7x
Infection	0001.xx-018.xx, 030.xx-041.xx, 050.xx-057.xx, 110.xx-118.xx, 070.xx-079.xx, 130.xx-136.xx, 400.xx-486.xx, 045.xx-049.xx, 995.91, 995.92
Injection site reaction	999.39
Leukopenia	288.8, 288.9
Elevated liver enzymes (AST, ALT)	790.4
Myalgia	729.1
Nausea/vomiting	780.0x, 780.52
Neutropenia	288.0x
Peripheral neuropathy	337.0x, 337.1, 356.x, 357.x
Pharyngitis	462
Pyrexia	780.6x
Rash	693.0, 708.8, 708.9, 782.1
Stomatitis	528.0x
Thrombocytopenia	287.4, 99.05

ICD-9 International Classification of Diseases version 9.

Total costs were tabulated from a payer perspective and included costs related to hospitalization, outpatient services, emergency department visits, chemotherapy and other (non-chemotherapy) medications.

### Statistical analyses

Mean AE counts and healthcare costs were calculated for each treatment cohort. Costs were adjusted to 2011 US dollars using the Medical Care Component of the U.S. Consumer Price Index (US Dept. of Labor and Bureau of Labor Statistics). In order to account for variation in the length of treatment episodes, average monthly costs were calculated by dividing the total costs incurred during the treatment episode by the episode length, in months. We evaluated average total costs as well as average costs categorize by resource type (hospitalization, outpatient services, emergency department visits, chemotherapy and other medications). The incremental monthly healthcare costs associated with the presence of AEs, compared with no AEs, were estimated using generalized linear models, controlling for age (linear) and Charlson Comorbidity Index (categorized as 0, 1, 2, and 3+) at study index date. The incremental cost associated with increasing the number of AEs was also estimated using similar generalized linear models. All analyses were performed using SAS version 9.3 (SAS Institute, Inc., Cary, NC) (SAS Institute, Inc 2012).

## Results

We identified 15,443 women with mBC that met inclusion criteria and were treated FL or SL with either a taxane or capecitabine (Figure 1). Mean age was approximately 50 years in all treatment cohorts (Table 2). Taxanes were used more frequently than capecitabine in both FL treatment (n = 7,934 vs. 634, respectively) and SL treatment (n = 6,339 vs. 360, respectively; Table 2). The mean length of FL treatment episodes was 161 days for regimens containing taxanes and 186 days for regimens containing capecitabine. Treatment duration was slightly shorter for SL, with a mean of 98 days for taxanes and 145 days for capecitabine. The mean length of treatment was significantly longer in patients who experienced at least one AE compared to those with no AEs in both FL and SL regimens (p <0.001 for each, Table 2). During FL treatment, 83.8% of taxane users and 45.1% of capecitabine users experienced at least one AE (p <0.0001). Adverse events were slightly less common in SL treatment, occurring in 68.0% of taxane users and 42.2% of capecitabine users (p <0.0001). Anemia was the most frequently diagnosed AE for all treatment cohorts, occurring in 20.5% of FL taxane users, 12.9% of FL capecitabine users, 16.0% of SL taxane users and 13.6% of SL capecitabine users (Table 2). Other commonly observed AEs included arthralgia, dyspnea, fatigue, infection and pyrexia.

**Table 2:** **Cohort characteristics, adverse event frequencies and costs**

	1st Line Taxane	1st Line Capecitabine	2nd Line Taxane	2nd Line Capecitabine
Number of patients	7934	634	6339	360
Age, mean (years)	49.3	51.8	48.4	50.1
Days per treatment episode, mean (SD)^‡^				
With no adverse events	128 (110)**	150 (123)**	91 (71)**	118 (95)**
With any adverse event	167 (134)	231 (209)	101 (98)	183 (156)
**Adverse events, n (%)**				
Any adverse event^†^	6647 (83.8)	286 (45.1)**	4313 (68.0)	152 (42.2)**
Liver enzymes increased	5 (0.1)	0 (0.0)	1 (0.0)	0 (0.0)
Anemia	1627 (20.5)	82 (12.9)	1017 (16.0)	49 (13.6)
Arthralgia	606 (7.6)	78 (12.3)	367 (5.8)	23 (6.4)
Bilirubin elevated	2 (0.0)	0 (0.0)	2 (0.0)	0 (0.0)
Constipation	0 (0.0)	0 (0.0)	0 (0.0)	0 (0.0)
Diarrhea	500 (5.0)	25 (3.9)	124 (1.9)	9 (2.5)
Dyspnea	733 (9.2)	62 (9.8)	394 (6.2)	27 (7.5)
Edema	331 (4.2)	21 (3.3)	199 (3.1)	3 (0.8)
Fatigue	817 (10.3)	43 (6.8)	493 (7.8)	26 (7.2)
Infection	750 (9.4)	39 (6.1)	354 (5.6)	29 (8.1)
Injection site reactions	4 (0.1)	0 (0.0)	0 (0.0)	0 (0.0)
Leukopenia	40 (0.5)	0 (0.0)	29 (0.5)	1 (0.3)
Myalgia	171 (2.2)	8 (1.3)	105 (1.6)	3 (0.8)
Nausea/vomiting	206 (2.6)	13 (2.0)	113 (1.8)	12 (3.3)
Neutropenia	203 (2.6)	2 (0.3)	12 (0.2)	0 (0.0)
Peripheral neuropathy	195 (2.5)	18 (2.8)	261 (4.1)	10 (2.8)
Pharyngitis	181 (2.3)	10 (1.6)	100 (1.6)	5 (1.4)
Pyrexia	929 (11.7)	27 (4.3)	247 (3.9)	14 (3.9)
Rash	312 (3.9)	17 (2.7)	161 (2.5)	7 (1.9)
Stomatitis	101 (1.3)	2 (0.3)	32 (0.5)	2 (0.6)
Thrombocytopenia	64 (0.8)	3 (0.5)	6 (0.1)	0 (0.0)
**Unadjusted monthly costs, mean (SD)** ^†^				
Chemotherapy costs	$6,256 (4659)	$3,761 (3221)**	$3,062 (4096)	$4,344 (3380)**
Other medication costs	$312 (794)	$279 (651)	$268 (873)	$281 (560)
Hospitalization costs	$817 (4084)	$1,065 (6159)	$490 (2826)	$1,655 (7324)*
Emergency department costs	$90 (696)	$46 (180)**	$99 (691)	$68 (312)*
Outpatient services costs	$293 (2607)	$1,220 (4465)**	$7,021 (5720)	$4,032 (6826)**
Total costs	$7,770 (6880)	$6,372 (8313)**	$10,939 (8145)	$10,381 (11139)

SD standard deviation, ^‡^p-values compare those with no AEs vs. those with AEs, ^†^p-values compare taxane vs. capecitabine treated patients for each treatment line, *p < 0.05, **p < 0.0001.

Unadjusted mean total monthly costs ranged from $6,372 to $10,939, depending on the treatment cohort (Table 2). Costs were higher in those treated with taxanes than in those treated with capecitabine, in both FL and SL settings. This difference was significant in those receiving FL treatment (p <0.0001) and was driven by chemotherapy costs in this cohort.

Adjusted total monthly costs were also significantly higher in those who experienced at least one AE than in those without AEs, both in FL (incremental cost: taxanes $1,142, p < 0.0001; capecitabine $1,817, p < 0.05) and SL (incremental cost: taxanes $1,448, p < 0.0001; capecitabine $4,437, p < 0.05) settings. In addition, total adjusted incremental costs increased as the number of AEs increased for all treatment cohorts (Table 3). The occurrence of AEs had the greatest and most consistent impact on hospitalization costs, which were significantly higher for those experiencing at least one AE and increased with the number of AEs in all treatment cohorts (Table 3). Chemotherapy costs were also higher for those experiencing AEs, though this trend was not significant for the SL capecitabine group. The impact of AEs on other cost categories differed across treatment cohorts. For example, among individuals treated FL or SL with capecitabine, costs related to other medications and ER visits were significantly higher in patients experiencing AEs than in those without AEs. However, for those receiving taxanes, costs of non-chemotherapy medications were lower while ER visit costs were not significantly different in patients experiencing AEs compared to those without AEs (Table 3). In the FL cohorts, total costs increased, despite decreases in outpatient costs.

**Table 3:** **Regression estimated average incremental monthly costs by adverse event count** ^**‡**^

	1st Line Taxane	1st Line Capecitabine	2nd Line Taxane	2nd Line Capecitabine
**With any adverse event, $**				
Chemotherapy costs	$478**	$630*	$513**	$118
Other medication costs	-$5	$176*	-$54*	$139*
Hospitalization costs	$920**	$1,745*	$441**	$2,771*
Emergency department costs	$29	$43*	$17	$91*
Outpatient services costs	-$280**	-$777*	$531*	$1,319
Total costs	$1,142**	$1,817*	$1,448**	$4,437*
**With 1–2 adverse events, $**				
Chemotherapy costs	$342*	$75	$22	$1
Other medication costs	-$3	$151*	-$13	$52
Hospitalization costs	$169	$755	$57	$1,018
Emergency department costs	-$6	$25	$23	$34
Outpatient services costs	-$226*	-$903*	$154	-$19
Total costs	$276	$104	$243	$1,087
**With 3–4 adverse events, $**				
Chemotherapy costs	$549*	$551	$325*	-$283
Other medication costs	$14	$220*	-$101*	$260*
Hospitalization costs	$296	$1,648	$183	$1,221
Emergency department costs	-$12	$50	-$3	$174*
Outpatient services costs	-$182	-$817	$1,103**	$693
Total costs	$667*	$1,652	$1,506**	$2,065
**With greater than 4 adverse events, $**				
Chemotherapy costs	$488*	$1,537**	$1,053**	$535
Other medication costs	-$17	$192*	-$71*	$191*
Hospitalization costs	$1,389**	$3,393**	$934**	$5,777**
Emergency department costs	$54*	$67*	$24	$128*
Outpatient services costs	-$353**	-$555	$593*	$3,323*
Total costs	$1,562**	$4,635**	$2,533**	$9,954**

^‡^Relative to those with no adverse events and adjusted for age (linear) and Charlson Comorbidity Index (categorized 0, 1, 2, and 3+), *p < 0.05, **p < 0.0001.

## Discussion

Metastatic breast cancer imparts a substantial economic burden on patients and society. We found that mean total monthly healthcare costs for mBC patients treated with a taxane or capecitabine ranged from $6,372 to $10,939, depending on the treatment cohort. These results are similar to those from a previously published study, which found that the average total healthcare cost in mBC patients receiving chemotherapy was $161,816 over a mean follow-up time of 532 days, or $9,252 per month (adjusted to 2011 US dollars) (Vera-Llonch et al. 2011). Results of the present study also demonstrate that experiencing any chemotherapy-related AEs increased monthly healthcare costs by $1,142 to $4,437. This is a considerable economic impact, even relative to the overall cost of healthcare in these patients. Furthermore, a trend was observed in which costs increased as the number of AEs increased. The incremental cost of AEs was primarily driven by greater hospitalization rates amongst those experiencing AEs. This aligns with results from previous studies, which found that cost increases associated with chemotherapy-related AEs were related to increases in pharmacy and hospitalization costs (Guerin et al. 2011; Hurvitz et al. 2012).

It was interesting to note that those with AEs also had greater chemotherapy costs than those without AEs across all treatment cohorts. This suggests that experiencing AEs may be a marker of greater chemotherapy exposure. In other words, those with the greatest treatment persistence would both incur greater chemotherapy costs and be more likely to experience AEs. This was further supported by the finding that treatment duration was significantly longer in those experiencing AEs than in those without AEs in both FL and SL regimens. However, this issue is not straightforward. Because chemotherapy-related AEs can result in treatment interruptions and dose reductions, we might conversely expect that those with AEs would receive less chemotherapy and therefore have lower chemotherapy costs. Our study allowed for breaks in therapy, only terminating treatment episodes if there was a gap in therapy of at least 45 days. However, shorter AE-related treatment gaps could exist within a single treatment episode. Most likely, both of these factors are operating in our study population simultaneously.

We found that capecitabine-based regimens were associated with fewer AEs, and lower costs, than taxane-based regimens. This was true in both FL and SL treatment cohorts, though the difference was only significant in those treated FL. We would expect that superior tolerability might improve patient adherence and persistence. This is supported by the finding that treatment-episodes in those treated with capecitabine were longer, on average, than in those treated with a taxane, in both FL and SL settings. However, it is interesting to note that incremental costs associated with AEs are numerically higher in those treated with capecitabine than in those treated with taxanes. It is possible that, while capecitabine treatment is associated with fewer AEs and lowers costs overall, the AEs experienced by capecitabine-treated individuals are of a greater severity or longer duration than in those treated with taxanes. Yet, with the current study we are unable to ascertain the severity or length of AEs.

Finally, our results indicate that AEs are less common in patients treated SL than in those receiving FL treatment. Perhaps the most likely explanation for this is that FL treatment episodes were longer, on average, than SL treatment episodes, which means that FL patients had more time in which to experience an AE.

### Limitations

Though healthcare claims databases provide rich information for analyzing healthcare utilization and costs, this type of data has a number of limitations. First, we were dependent on healthcare claims for the diagnosis and treatment information used to identify and classify patients. Furthermore, information on certain potential confounding variables, such as disease severity, was unavailable and, thus, could not be included in regression models. The degree to which we could look back historically was also limited. For these reasons, patients may have been erroneously included in or excluded from the study or incorrectly assigned to treatment cohorts. In addition, small sample sizes prevented us from analyzing the impact of individual AEs on costs. We also relied upon diagnosis codes to indicate the presence of AEs. Adverse events were only captured if they (1) resulted in a service in which a specific diagnosis was made, or (2) required a specified confirmatory laboratory test. Moreover, patients in this study were treated in a community setting, which differs substantially from clinical trials, where events of every severity are documented. Perhaps as a result, adverse event rates in this study were generally lower than those observed in the clinical trials for these agents (Blum et al. 1999; Jones et al. 2005). For example, though package inserts report frequencies of nausea and vomiting exceeding 30% for both taxanes and capecitabine, these events were only diagnosed in 1.8 to 3.3% of patients in this study (Taxotere 2013; Taxol 2011; Xeloda 2011). The undetected AEs were probably of lower severity, and would, therefore, only modestly impact costs. However, out of pocket payments were also excluded, since only those costs that were paid by employer-based insurance plans participating in Marketscan were captured. Given the high probability that we omitted at least some AEs and costs, the results of the present study likely underestimate the true cost of chemotherapy-related AEs in this population. Finally, because Medicare data were not included in this analysis, the results are only applicable to the population of mBC patients under age 65.

## Conclusions

Adverse events are common in women with mBC receiving taxane- and capecitabine-based chemotherapy regimens. These AEs may impact treatment dose and duration and are also associated with substantial increases in costs for both FL and SL treatment regimens. Clinicians and patients should consider the impact of AEs on costs and outcomes when selecting treatment options for women with mBC.

### Transparency

- Research support: This study was sponsored by Genentech, Inc.
- Research was previously presented at the ASCO Quality of Care Conference: Hansen RN, Ramsey SD, Lalla D, Masaquel A, Brammer M, Hurvitz S, Sullivan SD. The Cost of Adverse Events in Metastatic Breast Cancer in Taxane and Capecitabine Based Regimens. American Society of Clinical Oncology Quality Care Symposium. November 2, 2013, San Diego, CA.
